# Salivary asprosin, IL-39, IL-40, and IL-1β levels in diabetic patients with periodontitis: A cross-sectional analysis

**DOI:** 10.1007/s00784-026-06744-8

**Published:** 2026-01-14

**Authors:** Ayse Humeyra Oruc, Osman Babayiğit

**Affiliations:** Faculty of Dentistry, Department of Periodontology, Necmettin Erbakan University, Konya, 42090 Turkey

**Keywords:** Adipokine, Asprosin, BMI, Diabetes mellitus, HbA1c, Periodontitis, Saliva

## Abstract

**Objective:**

Periodontitis and type 2 diabetes mellitus (T2DM) are chronic diseases with a well-established bidirectional relationship. Recent studies have focused on salivary biomarkers to better understand their shared inflammatory mechanisms. This study aimed to assess salivary levels of asprosin, interleukin (IL) -39, IL-40, and IL-1β in patients with and without periodontitis and/or diabetes.

**Materials and methods:**

Eighty-eight participants were classified into four groups based on periodontal and diabetic status. Unstimulated saliva samples were collected and analyzed by ELISA. In addition to clinical periodontal parameters, body mass index (BMI) and glycated hemoglobin (HbA1c) levels were recorded.

**Results:**

Salivary asprosin and IL-1β levels were elevated in periodontitis groups regardless of T2DM status and showed positive correlations with BMI. IL-39 showed no significant findings. All biomarkers, with the exception of IL-39, were positively correlated with clinical periodontal parameters. IL-40 was elevated in both diabetic and non-diabetic patients with periodontitis.

**Conclusions:**

Elevated salivary asprosin, IL-40, and IL-1β levels in diabetic individuals with periodontitis may indicate their involvement in the inflammatory interactions linking metabolic dysfunction and periodontal tissue breakdown. IL-39 showed limited utility. These findings enhance understanding of salivary inflammatory patterns in diabetes-associated periodontitis, although the saliva-only, cross-sectional design warrants cautious interpretation.

**Clinical relevance:**

The identification of salivary asprosin, IL-40, and IL-1β as potential indicators of periodontal and metabolic inflammation suggests that saliva-based testing could support non-invasive screening and monitoring in patients with diabetes and periodontitis.

**Clinical trial number:**

NCT06735313.

## Introduction

Periodontitis is a chronic, immune-inflammatory disease affecting the supporting structures of the teeth, initiated by dysbiotic microbial dental biofilms. The World Health Organization’s 2021 report indicates that over 3.5 billion persons were affected by oral diseases in 2017, with severe periodontitis impacting over 796 million people [1]. Although dental biofilm constitutes the primary etiologic factor, the susceptibility, progression, and severity of periodontitis are profoundly influenced by host-related factors, particularly systemic diseases [2]. Type 2 diabetes mellitus (T2DM), characterized by insulin resistance and β-cell dysfunction, is a major systemic comorbidity that alters immune responses, impairs neutrophil function, and elevates proinflammatory cytokines, thereby accelerating periodontal tissue destruction. Evidence supports a bidirectional relationship between T2DM and periodontitis [3]. The systemic inflammatory burden generated by periodontitis has been implicated in worsening glycemic control by increasing insulin resistance and circulating inflammatory mediators such as interleukin (IL)−1β, IL-6, tumor necrosis factor-alpha, and emerging molecules like IL-39, IL-40, and asprosin [4–7].

Given the limitations of conventional clinical and radiographic assessments in detecting active periodontal disease and predicting progression, salivary biomarkers have gained attention as a noninvasive tool for improving diagnostic accuracy [8]. Cytokines, as peptide-based signaling molecules, reflect both local and systemic inflammatory activity and therefore serve as useful indicators for understanding disease-associated immune responses [9, 10].

White adipose tissue functions not only as an energy store but also as an endocrine organ, producing adipokines that regulate inflammation and metabolism [11]. Asprosin, first described by Romere et al., is a recently identified glucogenic adipokine secreted by white adipose tissue, playing key roles in glucose homeostasis, appetite regulation, and pancreatic β-cell inflammatory processes [12]. Asprosin levels have been associated with obesity, and studies have reported elevations in individuals with periodontitis, suggesting a potential link between metabolic dysregulation and periodontal inflammation [6].

IL-39 and IL-40 are novel cytokines with emerging relevance in chronic inflammatory diseases [13, 14]. IL-39, a member of the IL-12 family, is produced by LPS-stimulated B cells and may promote leukocyte adhesion and transmigration via STAT1/3 signaling [15]. Evidence suggests that IL-39 has pro-inflammatory properties, as elevated levels have been reported in autoimmune and inflammatory disorders, including ankylosing spondylitis, neuromyelitis optica, and lupus-like models, where its inhibition reduced disease severity [16]. IL-40, encoded by the C17orf99 gene, is a B cell–associated cytokine involved in immune regulation and is also produced by other immune cells, including CD68⁺ macrophages and CD4⁺/CD8⁺ T lymphocytes [4]. Elevated IL-40 levels have been detected in serum and salivary gland tissues of patients with primary Sjögren’s syndrome and in the serum of individuals with rheumatoid arthritis, suggesting a role in sustaining chronic inflammation [14, 17].

IL-1β is a well-established key proinflammatory cytokine associated with periodontal inflammation and bone resorption by inducing inflammatory mediators and matrix-degrading enzymes [18]. IL-1β, primarily produced by macrophages, monocytes, and neutrophils and elevated levels have also been linked to systemic conditions such as type 2 diabetes and obesity, where chronic low-grade inflammation contributes to disease progression [19].

This cross-sectional study focused on four salivary biomarkers selected for their complementary roles in inflammatory and metabolic pathways: asprosin as a glucogenic adipokine linked to insulin resistance; IL-39 and IL-40 as emerging IL-12 family–related cytokines associated with autoimmune and cardiometabolic inflammation; and IL-1β as a canonical pro-inflammatory cytokine and benchmark periodontal biomarker. Using this panel, we aimed to evaluate their salivary levels in individuals with and without periodontitis and/or T2DM and to examine their relationships with clinical periodontal parameters, body mass index (BMI), and glycated hemoglobin (HbA1c). The primary objective was to determine whether these molecules can non-invasively differentiate periodontal health from disease while considering diabetic status, and the secondary objective was to assess their association with metabolic control and to explore whether T2DM provides additional diagnostic value beyond periodontitis.

## Materials and methods

### Ethical Approval

This study was conducted following approval by the Ethics Committee for Non-Drug Clinical Research of the Faculty of Dentistry at Necmettin Erbakan University (Approval No: 2024/442, dated May 30, 2024) and was carried out in accordance with the tenets of the Declaration of Helsinki. All participants provided written and verbal informed consent prior to enrollment. This study is registered at ClinicalTrials.gov (Identifier: NCT06735313).

### Study Groups

A total of 88 individuals who presented to the Department of Periodontology, Faculty of Dentistry, Necmettin Erbakan University between November 2024 and January 2025 were included in this study. Participants were divided into four groups: systemically healthy with gingival health (H-GH) (*n* = 22), systemically healthy with periodontitis (H-P) (*n* = 22), patients with T2DM and gingival health (D-GH) (*n* = 22), and patients with T2DM and periodontitis (D-P) (*n* = 22).

The periodontal status of each participant was assessed by a calibrated examiner (A.H.O.) in accordance with the 2017 World Workshop Classification of Periodontal and Peri-Implant Diseases and Conditions, based on both clinical and radiographic evaluations [20]. Individuals with no radiographic bone loss (RBL) or clinical attachment loss, bleeding on probing (BOP) < 10%, and probing depth (PD) < 3 mm were classified as having gingival health. Inclusion in the periodontitis groups required a diagnosis of generalized Stage II or III and Grade B or C periodontitis. These patients presented with at least four interdental sites showing clinical attachment level (CAL) ≥ 4 mm, PD ≥ 4 mm, and BOP ≥ 30%, with generalized involvement (> 30% of teeth affected) and radiographic bone loss involving at least 15% of the coronal third of the root. Tooth loss due to periodontitis did not exceed four teeth. Grading was determined using the bone loss-to-age ratio as the primary indicator of disease progression. Individuals with T2DM were upgraded to Grade B or Grade C depending on glycemic control: patients with HbA1c < 7% were assigned to Grade B, whereas those with HbA1c ≥ 7% were assigned to Grade C. Systemically healthy periodontitis patients with a bone loss-to-age ratio ≥ 0.25 were assigned to Grade B or C [21].

**Inclusion Criteria:**

- Aged between 18 and 60 years
- Diagnosed with stage II or III periodontitis based on clinical evaluation
- Presence of at least 20 natural teeth (excluding third molars)
- For diabetic groups: confirmed diagnosis of T2DM without other systemic diseases
- Willingness to participate and provide informed consent

**Exclusion Criteria:**

- Presence of autoimmune diseases (e.g., rheumatoid arthritis, familial mediterranean fever, ankylosing spondylitis, Behçet’s disease, psoriasis)
- Systemic diseases such as nephrotic syndrome, chronic renal failure, or cardiovascular diseases
- Tobacco or alcohol use
- Undergoing chemotherapy, radiotherapy, or immunosuppressive therapy
- Recent periodontal treatment (within the last 6 months)
- Antibiotic use within the last 6 months
- Pregnancy or breastfeeding

For participants in the H-GH and H-P groups, the absence of diabetes was verified through standardized medical history screening and confirmation via Turkey’s national electronic health record system (e-Nabız) [22]. With participants’ permission, their e-Nabız data—including previous diagnoses, fasting plasma glucose or HbA1c results, and antidiabetic medication history—were reviewed, and any individual with evidence of diabetes or dysglycaemia was excluded from the systemically healthy groups.

The diagnosis of T2DM for participants in the D-GH and D-P groups was established according to the American Diabetes Association (ADA) criteria and verified through recent laboratory findings (fasting plasma glucose and/or HbA1c) retrieved from e-Nabız [23]. BMI was calculated during the clinical examination (kg/m²). HbA1c values were obtained from the most recent endocrinology reports recorded in e-Nabız (within the last 1 month). For diabetic participants without a recent result, an updated HbA1c test was requested, and the latest value was documented on the day of saliva sampling to ensure up-to-date glycemic data.

Demographic and medical histories were collected for all participants. Periodontal clinical and radiographic examinations were completed, and saliva samples were collected the following day.

### Clinical measurements

All teeth, excluding third molars, were examined at four sites (mesial, distal, buccal, and lingual/palatal) using a Williams periodontal probe (Chicago, IL, USA). The recorded clinical parameters included the Plaque Index (PI), Gingival Index (GI), BOP, PD, and CAL [24]. All measurements were performed by a single calibrated examiner (A.H.O.). Calibration exercises were conducted prior to the study to assess intra-examiner reliability, yielding kappa values > 0.85 for all periodontal measurements, indicating excellent reproducibility. Additionally, panoramic radiographs were obtained from all participants to evaluate alveolar bone levels and determine the stage and grade of periodontitis according to the current classification.

### Collection of Saliva Specimens

Unstimulated whole saliva samples were collected between 09:00 and 10:00 a.m., one day after clinical measurements, using the passive drool technique [25]. Participants were instructed to refrain from eating, drinking, chewing gum, smoking, brushing teeth, or using mouthwash for at least 2 h prior to sampling. Each participant was seated comfortably with the head slightly tilted forward, allowing saliva to pool at the floor of the mouth and expectorating into a graduated sterile polypropylene tube every 60 s. Collection continued until approximately 5 mL of unstimulated saliva was obtained. Samples were immediately placed on ice and centrifuged at 3000×g for 10 min at 4 °C following the protocol recommended for saliva processing [26]. The clear supernatant was carefully aspirated without disturbing the pellet and transferred into 1.5 mL Eppendorf tubes. If visible contamination (e.g., blood or food debris) was detected, the sample was recollected. Aliquots were stored at − 80 °C until analysis. All procedures were performed by the same calibrated examiner (A.H.O.) to ensure consistency and minimize pre-analytical variability.

### Measurement of asprosin, IL-39, IL-40, and IL-1β levels in saliva

Salivary levels of asprosin, IL-39, IL-40, and IL-1β were quantified using commercial ELISA kits (Bioassay Technology Laboratory, Shanghai Korain Biotech Co., Shanghai, China). Sample preparation, dilution, incubation, washing, substrate reactions, and absorbance measurements at 450 nm were carried out according to the manufacturer’s protocols using a microplate reader (Synergy HT, Biotek Instruments, USA). All procedures were conducted under sterile conditions. Detection ranges for the asprosin (E4095Hu), IL-39 (E7444Hu), IL-40 (E4654Hu), and IL-1β (E0143Hu) kits were 0.5-100ng/ml, 2-600ng/L, 1.5-96ng/ml, and 20-6000pg/mL, respectively.

### Statistical Analysis

Sample size was determined based on a previous case–control study by Gül et al. [6], which investigated salivary asprosin levels in 65 systemically healthy participants. Using the reported effect size, an a priori power analysis (G*Power v3.1.9.2; α = 0.05, 1 – β = 0.80) for four independent groups indicated a required total of 88 participants (22 per group), which was achieved in this study.

All statistical analyses were performed using IBM SPSS v27 (IBM Corp., Armonk, NY, USA). Descriptive statistics included mean, standard deviation, median, and range for continuous variables, and frequency and percentage for categorical variables. Normality was assessed with the Shapiro-Wilk test. Between-group comparisons were performed using Mann-Whitney U, one-way ANOVA, or Kruskal-Wallis H tests, as appropriate. Post-hoc analyses were conducted using Bonferroni or Dunn tests. Correlations were evaluated using Spearman correlation coefficients, interpreted as weak (0–0.29), moderate (0.30–0.70), or strong (0.71–1.0). ROC analysis was used to determine cut-off values, sensitivity, specificity, and area under the curve (AUC) for each biomarker. A p-value < 0.05 was considered statistically significant.

## Results

### Demographic features

The distribution of demographic variables is presented in Table 1. There were no statistically significant differences among the groups in terms of gender or age (*p* = 0.746 and *p* = 0.506, respectively). BMI was highest in the D-GH group and lowest in the H-GH group, with statistically significant differences observed between the H-GH group and all others (*p* < 0.001). The mean HbA1c was significantly higher in the D-P group (8.47 ± 1.94) compared to the D-GH group (6.55 ± 1.74) (*p* < 0.001).

**Table 1:** Comparison of demographic characteristics, BMI, and HbA1c levels among study groups

		H-GH	H-*P*	D-GH	D-*P*	Total	*p*
Gender	Female (n %)	13 (59.1)	12 (54.5)	10 (45.5)	10 (45.5)	45 (51.1)	0.746*
	Male (n %)	9 (40.9)	10 (45.5)	12 (54.5)	12 (54.5)	43 (48.9)	
Age	Mean ± SD	43 ± 3.5	43.4 ± 8.3	46 ± 12.1	45.8 ± 5.2	44.6 ± 8	0.506***
	Median (min-max)	43(35–49)	41.5(30–65)	46(24–68)	47(33–53)	45(24–68)	
BMI	Mean ± SD	23.95 ± 3.78**ᵃ**	27.924 ± 4.99**ᵇ**	29.41 ± 3.8**ᵇ**	28.36 ± 4.41**ᵇ**	27.41 ± 4.69	< 0.001***
	Median (min-max)	22.44 (18.42–31.74)	28.17 (19.47–41.52)	29.35 (23.43–36.85)	27.54 (21.16–38.67)	27.26 (18.42–41.52)	
HbA1c	Mean ± SD	.	.	6.55 ± 1.74**ᵃ**	8.47 ± 1.94**ᵇ**	7.51 ± 2.06	< 0.001**
	Median (min-max)	.	.	6.04(5.04–11.09)	8.07 (6.03–12.03)	7.03 (5.04–12.04)	

Groups sharing the same superscript letter do not differ significantly. H-P: systemically healthy individuals with periodontitis; H-GH: systemically healthy individuals with gingival health; D-GH: individuals with diabetes and gingival health; D-P: individuals with diabetes and periodontitis; SD: standard deviation; BMI: body mass index; HbA1c: glycated hemoglobin. * Pearson Chi-square test and Fisher–Freeman–Halton exact test; ** Mann–Whitney U test; *** Kruskal–Wallis test

### Clinical periodontal parameters

The full-mouth clinical periodontal parameters of the study groups are summarized in Table 2. Statistically significant differences were observed among all groups for BOP%, PD, CAL, GI, and PI (*p* < 0.001). Periodontitis groups (H-P and D-P) had significantly higher clinical periodontal parameter values than healthy groups (H-GH and D-GH) (*p* < 0.001).

**Table 2:** Comparison of full-mouth periodontal parameters among study groups

Clinical periodontal parameters		H-GH (*n* = 22)	H-*P* (*n* = 22)	D-GH (*n* = 22)	D-*P* (*n* = 22)	Total (*n* = 88)	*p*
BOP%	Mean ± SD	4.48 ± 2.94ᵃ	80.72 ± 11.5ᵇ	5.39 ± 3.05ᵃ	76.27 ± 11.52ᵇ	41.71 ± 37.94	**< 0.001**
	Median(min-max)	4.9(0–8)	82.4(50–100)	6.5(0–9.3.3)	75(53.5–100)	29.65(0–100)	
PD (mm)	Mean ± SD	1.8 ± 0.41ᵃ	3.53 ± 0.63ᵇ	1.57 ± 0.34ᵃ	3.69 ± 1.06ᵇ	2.65 ± 1.18	**< 0.001**
	Median(min-max)	1.85(1.2–2.7)	3.35(2.8–5.1)	1.65(1–2.1.1)	3.4(2–7)	2.5(1–7)	
CAL (mm)	Mean ± SD	0.18 ± 0.5ᵃ	4.38 ± 0.71ᵇ	0.32 ± 0.57ᵃ	4.54 ± 1.14ᵇ	2.36 ± 2.25	**< 0.001**
	Median(min-max)	0(0–2)	4.05(3–6.2.2)	0(0–2)	4.3(3.5–9.5)	2.5(0–9)	
GI	Mean ± SD	1.05 ± 0.21ᵃ	1.91 ± 0.53ᵇ	1.05 ± 0.21ᵃ	1.95 ± 0.38ᵇ	1.49 ± 0.57	**< 0.001**
	Median(min-max)	1(1–2)	2(1–3)	1(1–2)	2(1–3)	1(1–3)	
PI	Mean ± SD	1 ± 0ᵃ	2.27 ± 0.7ᵇ	1.32 ± 0.48ᵃ	2.59 ± 0.59ᵇ	1.8 ± 0.83	**< 0.001**
	Median(min-max)	1(1–1)	2(1–3)	1(1–2)	3(1–3)	2(1–3)	

a-b: Groups sharing the same letter are not significantly different. H-GH: systemically healthy with gingival health, H-P: systemically healthy with periodontitis, D-GH: diabetic with gingival health, D-P: diabetic with periodontitis, BOP%: bleeding on probing percentage, PD: probing depth, CAL: clinical attachment level, GI: gingival index, PI: plaque index. Kruskal–Wallis and one-way ANOVA tests

### Comparison of salivary Asprosin, IL-39, IL-40, and IL-1β levels among groups

As shown in Table 3, salivary IL-1β, IL-40, and asprosin levels differed significantly among the groups (*p* < 0.001), while no significant difference was observed for IL-39 (*p* = 0.430). IL-1β levels were significantly elevated in all groups with periodontitis (H-P, D-P) and in diabetic individuals without periodontitis (D-GH) compared to the H-GH group. IL-40 levels were significantly higher in both D-P and H-P groups than in H-GH, with intermediate values in the D-GH group. Salivary asprosin levels were significantly higher in the H-P, D-GH, and D-P groups compared to the H-GH group (*p* < 0.001).

**Table 3:** Comparison of salivary Asprosin, IL-39, IL-40, and IL-1β levels among study groups

Salivary biomarkers		H-GH (*n* = 22)	H-*P* (*n* = 22)	D-GH (*n* = 22)	D-*P* (*n* = 22)	Total (*n* = 88)	*p*
IL-1β (pg/ml)	Mean ± SD	1011.59 ± 239.49**ᵃ**	1529.54 ± 234.91**ᵇ**	1419.72 ± 356.31**ᵇ**	1601.72 ± 241.79**ᵇ**	1390.64 ± 353.05	**< 0.001**
	Median (min-max)	1022.61 (332–1573)	1559.31 (1226–1971)	1359.36 (998–2349)	1536.05 (1281–2168)	1379.33 (332–2349)	
IL-39 (pg/ml)	Mean ± SD	158.75 ± 83.67	122.02 ± 21.48	142.64 ± 67.54	116.34 ± 24.13	134.94 ± 57.71	0.430
	Median (min-max)	130.51 (76.63–361.39.63.39)	122.20 (81.38–166.64.38.64)	122.60 (62.88–359.60)	106.74 (84.82–188.72.82.72)	122.46 (62.88–361.39.88.39)	
IL-40 (ng/ml)	Mean ± SD	27.82 ± 8.7**ᵃ**	35.53 ± 5.2**ᵇ**	32.69 ± 9.1**ᵃᵇ**	36.73 ± 5.5**ᵇ**	33.195 ± 8.0	**< 0.001**
	Median (min-max)	25.3 (11.3–44.3)	36.0 (26.0–47.3.0.3)	30.4 (16.6–52.2)	37.7 (23.5–45.7)	33.3 (11.3–52.2)	
Asprosin (ng/ml)	Mean ± SD	22.08 ± 6.1**ᵃ**	29.83 ± 6.1**ᵇ**	26.71 ± 4.78**ᵇ**	30.82 ± 4.6**ᵇ**	27.36 ± 6.3	**< 0.001**
	Median (min-max)	23.24 (10.72–31.55)	29.66 (20.64–43.97)	27.61 (15.47–34.04)	30.29 (22.45–40.73)	27.73 (10.72–43.97)	

Different superscript letters indicate statistically significant differences between groups. Groups sharing at least one common letter (e.g. ‘a’ and ‘ab’ or ‘b’ and ‘ab’) are not significantly different from each other. H-GH: systemically healthy with gingival health, H-P: systemically healthy with periodontitis, D-GH: diabetic with gingival health, D-P: diabetic with periodontitis, pg/ml: picograms per milliliter, ng/ml: nanograms per milliliter, SD: standard deviation, min-max: minimum–maximum values. Kruskal–Wallis test and one-way ANOVA test

### Correlations

In Table 4, Spearman correlation analysis revealed a weak but statistically significant positive correlation between BMI and salivary asprosin levels (*r* = 0.284, *p* < 0.01). Asprosin levels also showed moderate positive correlations with various clinical periodontal parameters, including BOP%, PD, CAL, GI, and PI (r values ranging from 0.373 to 0.467; *p* < 0.01). Among all biomarkers, IL-1β demonstrated the strongest associations with periodontal parameters, particularly with CAL (*r* = 0.611), PD (*r* = 0.499), and PI (*r* = 0.585) (*p* < 0.01 for all). In contrast, IL-39 showed a weak and inverse correlation only with HbA1c (*r* = − 0.309, *p* < 0.05), and did not exhibit significant associations with periodontal parameters. IL-40 showed moderate correlations with BOP%, PD, CAL, GI, and PI (*r* = 0.344–0.459, *p* < 0.01), suggesting its potential role in periodontal inflammation.

**Table 4:** Spearman correlation coefficients among BMI, HbA1c, periodontal Parameters, and salivary biomarkers

	BMI	HbA1c	IL-1β	IL-39	IL-40	Asprosin
BMI	1	−0.172	0.349**	−0.035	0.19	0.284**
HbA1c	−0.172	1	0.173	−0.309*	0.134	0.199
BOP%	0.094	0.464**	0.478**	−0.150	0.344**	0.373**
PD	0.120	0.488**	0.499**	−0.086	0.421**	0.381**
CAL	0.146	0.507**	0.611**	−0.051	0.459**	0.431**
GI	0.116	0.439**	0.477**	−0.018	0.433**	0.397**
PI	0.262*	0.451**	0.585**	−0.105	0.374**	0.467**

*: *p* < 0.05, **: *p* < 0.001; BMI: Body mass index, HbA1c: Glycated hemoglobin, BOP%: Bleeding on probing percentage, PD: Probing depth, CAL: Clinical attachment level, GI: Gingival index, PI: Plaque index. Spearman correlation test

### ROC analysis

**Fig. 1:**
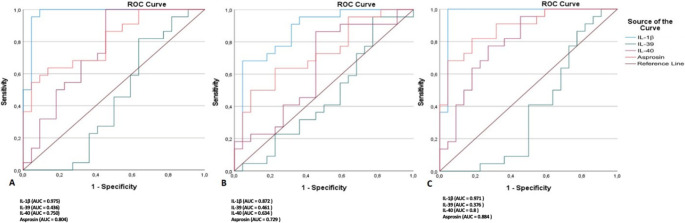
ROC curves illustrating the diagnostic performance of salivary Asprosin, IL-39, IL-40, and IL-1β in differentiating between study groups. **(A)** H-P vs. H-GH, **(B)** D-GH vs. H-GH, **(C)** D-P vs. H-GH

In Table 5, ROC curve analysis was performed to evaluate the diagnostic performance of salivary biomarkers in distinguishing the diseased groups (H-P, D-GH, and D-P) from the H-GH group (Fig. 1). IL-1β exhibited the highest discriminative performance across all comparisons, with AUC values ranging from 0.872 to 0.975 (*p* < 0.001), indicating excellent discriminative ability for both periodontitis and diabetes-related inflammation. Asprosin also showed moderate-to-high discriminative ability, particularly in the D-P vs. H-GH comparison (AUC = 0.884, sensitivity = 81.8%, specificity = 81.8%, *p* < 0.001). It also yielded significant results in the H-P and D-GH comparisons (AUC = 0.804 and 0.729, respectively). IL-40 demonstrated moderate discriminative ability in the D–P (AUC = 0.800) and H–P (AUC = 0.750) comparisons, but did not reach statistical significance in the D–GH group (*p* = 0.120). IL-39, on the other hand, showed low AUC values (0.376–0.461) with poor sensitivity and specificity, indicating limited diagnostic utility.

**Table 5:** Diagnostic performance of salivary biomarkers in distinguishing study groups from the H-GH group based on ROC curve analyses

Groups	Salivary Biomarkers	Cut-off	AUC	Sensitivity (%)	Specificity (%)	*p*
H-P vs. H-GH	IL-1ß	1228.5	0.975 (0.929–1.000.929.000)	95.5	95.5	< 0.001
	IL-39	106.5	0.436 (0.257–0.615)	81.8	36.4	0.467
	IL-40	25.5	0.750 (0.602–0.898)	100.0	54.5	0.005
	Asprosin	29.5	0.804 (0.676–0.931)	54.5	95.5	0.001
D-GH vs. H-GH	IL-1ß	1243	0.872 (0.767–0.977)	68.2	95.5	< 0.001
	IL-39	102	0.461 (0.286–0.636)	72.7	31.8	0.660
	IL-40	25	0.634 (0.465–0.804)	86.4	54.5	0.120
	Asprosin	26	0.729 (0.581–0.878)	63.6	77.3	0.003
D-P vs. H-GH	IL-1ß	1254	0.971 (0.914–1.000.914.000)	100.0	95.5	< 0.001
	IL-39	102	0.376 (0.202–0.550)	63.6	31.8	0.159
	IL-40	33.5	0.8 (0.667–0.932)	77.3	72.7	0.001
	Asprosin	26	0.884 (0.788–0.981)	81.8	81.8	< 0.001

AUC: Area under the curve; H-P: Systemically healthy with periodontitis; H-GH: Systemically healthy with gingival health,; D-GH: Diabetic with gingival health; D-P: Diabetic with periodontitis

## Discussion

This study investigated salivary levels of asprosin, IL-39, IL-40, and IL-1β in systemically healthy and diabetic individuals with or without periodontitis. To the best of our knowledge, this is the first study to simultaneously evaluate salivary asprosin, IL-39, and IL-40 in both periodontitis and diabetic patient groups, and specifically the first to quantify IL-39 in saliva. Patients with diabetic periodontitis showed elevated salivary concentrations of asprosin, IL-40, and IL-1β, indicating that these biomarkers may hold diagnostic relevance in metabolically driven inflammatory conditions.

In the current study, age and gender were balanced across groups. BMI was significantly higher in the diabetic groups than in the systemically healthy groups and was also higher in the H-P group compared to H-GH group. This is consistent with the established link between obesity, insulin resistance, and heightened systemic inflammation in individuals with T2DM, which may in turn exacerbate periodontal tissue destruction [27]. HbA1c levels were significantly higher in the D-P group compared to the D-GH group, supporting the idea that the presence of periodontitis may worsen glycemic control [28].

BOP%, PD, CAL, GI, and PI values were higher in the H-P and D-P groups compared with their healthy counterparts, as these parameters formed the basis for assigning individuals to the periodontitis categories. D-P group showed the most severe periodontal parameters, suggesting that diabetes may accelerate periodontitis progression by disrupting immune regulation. However, diabetes alone had a milder effect in the absence of periodontitis, likely influenced by glycemic control, disease duration, treatment efficacy, and individual immune variability [29]. When examining the correlations between clinical periodontal parameters and metabolic, BMI showed a weak positive correlation only with PI, suggesting a link between higher body mass and poorer oral hygiene. In contrast, HbA1c demonstrated moderate positive correlations with all clinical periodontal parameters (r values ranging from 0.439 to 0.507; *p* < 0.01). These findings support the hypothesis that poor glycemic control may contribute to increased probing pocket depth, clinical attachment loss, and alveolar bone resorption, independently of obesity status [30]. Also, no significant correlation was found between BMI and HbA1c, likely due to variability in glycemic control among diabetic participants. This aligns with NHANES-based studies reporting no significant association between BMI and HbA1c in diabetic individuals [31].

Given the complex interplay between metabolic status and inflammation, attention has turned to emerging salivary biomarkers such as asprosin, a peptide hormone predominantly secreted by white adipose tissue that plays crucial roles in energy metabolism and glucose homeostasis [12]. Elevated serum asprosin levels have been documented in T2DM patients [32]. Studies suggest that asprosin may have a role not only in metabolic regulation but also in inflammatory responses [33, 34]. Recent findings also confirmed the secretion of asprosin from human salivary glands, emphasizing saliva as a viable, non-invasive diagnostic medium [35]. Tutuş et al. reported elevated asprosin levels in saliva, gingival crevicular fluid (GCF), and serum of periodontitis patients [36]. Similarly, Gül et al. demonstrated positive correlations between serum asprosin concentrations, periodontal clinical parameters, and CRP levels in patients with periodontitis and acute myocardial infarction, underscoring its potential role as a biomarker linking periodontal and cardiovascular diseases [37]. Our study revealed significantly higher salivary asprosin levels in both periodontitis groups compared to healthy controls, with the highest levels in the D-P group. Elevated levels were also observed in diabetic individuals with gingival health, suggesting a link to systemic inflammation. Asprosin positively correlated with all clinical periodontal parameters and demonstrated the highest discriminative performance in the D–P group; however, these findings should be interpreted as exploratory rather than indicative of validated diagnostic capability.

Beyond the influence of T2DM, adiposity itself may modulate asprosin concentrations and increase periodontal inflammation, underscoring the need to evaluate its relationship with BMI [38]. Supporting this Sadeghi et al. reported that weight and BMI reduction improved glucose homeostasis and lowered serum asprosin levels, supporting its role in glycemic regulation [39]. Similarly, Gül et al. and Ugur & Aydin found higher asprosin concentrations in obese individuals, with positive correlations to BMI [6, 35]. Consistent with previous reports, our study also found a positive correlation between salivary asprosin and BMI, suggesting that adiposity may contribute to elevated asprosin levels and associated metabolic and periodontal inflammation.

IL-39, a recently identified cytokine formed by the heterodimerization of the IL-23 p19 subunit with the EBI3 molecule, is primarily produced by activated B cells and has been implicated in neutrophil activation and lupus-like inflammatory responses in murine models [13, 40]. Several studies have linked elevated serum IL-39 levels to acute coronary syndrome, suggesting its potential role as a biomarker in cardiovascular inflammation [15, 41]. Similarly, Sari et al. reported comparable findings in their study involving individuals without systemic diseases, where IL-39 concentrations were notably higher in those with periodontal inflammation [5]. Another study reported elevated GCF IL-39 levels in both periodontitis and diabetic patients, with the highest levels in those with both conditions [42]. Given these findings, the clinical relevance of IL-39 remains uncertain. Nussrat and Ad’hiah found no significant variation in IL-39 levels across HbA1c-based subgroups, suggesting independence from glycemic control [43]. Moreover, other reports have highlighted the limited and sometimes conflicting evidence regarding IL-39’s expression and biological activity in human immune cells [44–46]. In the context of autoimmune disorders, Weng et al. reported that individuals with Hashimoto’s thyroiditis and Graves’ disease had significantly lower IL-39 levels compared to healthy controls [47]. However, ROC curve analysis showed limited diagnostic utility of IL-39 in distinguishing these autoimmune conditions. Similarly, salivary IL-39 concentrations in the present study were highest in the H-GH group and decreased across all other clinical groups, and the weak negative correlation between IL-39 levels and HbA1c values also reflected the pattern described by Weng et al. [47]. Furthermore, ROC analyses revealed no significant diagnostic utility for IL-39 in distinguishing periodontitis or diabetes, as all AUC values remained well below the threshold of clinical relevance. This limited diagnostic performance may, at least in part, be attributable to the exclusive assessment of IL-39 in saliva, as its concentrations and detectability could differ across other biological fluids.

IL-40 is a newly described inflammatory mediator with emerging roles in autoimmune and inflammatory diseases; however, evidence regarding its involvement in periodontal pathology remains limited [17, 48]. Recent studies have reported elevated IL-40 levels in systemic conditions such as ankylosing spondylitis, rheumatoid arthritis, and T2DM, suggesting a link between IL-40 and systemic immune activation [4, 16, 17, 48]. A recent salivary study by Babun et al. demonstrated significantly increased IL-40 levels in gingivitis and periodontitis patients, with positive correlations to clinical periodontal parameters, indicating that IL-40 may respond early to inflammatory challenges in the oral cavity [49]. Consistent with these observations, the present study also identified elevated salivary IL-40 concentrations in both diabetic and non-diabetic periodontitis groups, accompanied by positive correlations with clinical periodontal parameters. Interestingly, even diabetic individuals with clinically healthy gingiva showed significantly higher IL-40 levels compared to H-GH group, supporting the hypothesis that IL-40 may be sensitive to subclinical inflammatory changes associated with metabolic dysregulation. This suggests that IL-40 is not merely a local marker of periodontal destruction but may also reflect systemic immunometabolic disturbances, particularly in the context of T2DM. Furthermore, ROC curve analyses in our study showed that IL-40 had moderate discriminative ability in distinguishing periodontal status, with stronger separation observed in diabetic individuals with periodontitis. The data collectively indicate that IL-40 may reflect the combined influence of metabolic and periodontal inflammatory processes.

IL-1β is a well-established proinflammatory marker in periodontitis, with levels rising in both local and systemic inflammation [50, 51]. Chronic systemic inflammation is a hallmark of T2DM and is caused by higher levels of pro-inflammatory cytokines like IL-1β, which make insulin resistance and β-cell dysfunction worse [52]. Our study aligns with previous literature, demonstrating significantly elevated salivary IL-1β levels in periodontitis groups compared to healthy controls, irrespective of diabetic status, and showing positive correlations with clinical periodontal parameters [19]. Salivary IL-1β was also significantly increased in diabetic individuals without periodontitis compared to systemically healthy individuals, suggesting diabetes alone might enhance inflammatory responses even in clinically healthy periodontal tissues. Although the D-P group exhibited both systemic and local inflammation, salivary IL-1β levels were not significantly higher than those in the H-P group. This may be explained by IL-1β’s role as an insulin antagonist, which inhibits insulin receptor tyrosine kinase activity (including autophosphorylation and phosphotransferase functions). Moreover, use of oral hypoglycemic agents or exogenous insulin in diabetic individuals can suppress IL-1β expression, potentially offsetting the additive inflammatory effect of diabetes [19].

Previous research indicated that higher BMI is associated with elevated IL-1β concentrations, underscoring the obesity’s low-grade chronic inflammation to intensify periodontal inflammation [7, 53]. Consistent with these findings, our study observed a positive correlation between BMI and salivary IL-1β, suggesting that metabolic status may influence its salivary expression. Furthermore, the ROC curve analysis revealed that salivary IL-1β demonstrated strong discriminative ability in distinguishing both diabetic and non-diabetic individuals with periodontitis from H-GH group. While these results are consistent with the established role of IL-1β in periodontal inflammation, they should be interpreted as preliminary and hypothesis-generating rather than as evidence of validated diagnostic cut-offs.

This study has several limitations that should be acknowledged. Its cross-sectional design precludes causal inference and prevents evaluation of temporal changes in biomarker levels or their response to periodontal therapy. Although the study was carefully designed, potential sources of bias and sampling error inherent to cross-sectional studies cannot be entirely excluded. Consecutive sampling, strict inclusion and exclusion criteria, examiner calibration, and an a priori power analysis were applied to minimize these effects. The relatively modest sample size may limit the generalizability of the findings. Moreover, uncontrolled lifestyle and environmental factors—such as diet, stress, socio-economic background, and oral hygiene practices—were not fully standardized and may have influenced salivary cytokine levels. In addition, only saliva samples were analyzed; the lack of corresponding serum or GCF measurements restricted the ability to compare local and systemic cytokine dynamics. Moreover, although all diabetic participants met ADA diagnostic criteria, the type of antidiabetic therapy (oral hypoglycemic agents and/or insulin), detailed information on diabetes duration and glycemic variability were not incorporated into the analyses, which may have influenced systemic and salivary cytokine responses. Finally, salivary flow rate was not measured, and biomarker concentrations were not normalized to flow. This may have influenced between-group comparisons and should be considered in future studies.

### Conclusions

This study demonstrated that salivary asprosin, IL-1β, and IL-40 levels were significantly elevated in diabetic individuals with periodontitis and were associated with clinical periodontal parameters. IL-39 showed limited utility. Asprosin also reflected metabolic characteristics through its associations with BMI and HbA1c. These findings indicate that the evaluated biomarkers may reflect the interplay between metabolic status and periodontal inflammation; however, no diagnostic inferences can be made given the saliva-only, cross-sectional design, and further studies incorporating systemic measurements are required to determine their clinical applicability.

## Data Availability

The datasets used and/or analysed during the current study are available from the corresponding author on reasonable request.
